# COVID-19-Associated Encephalopathy—Case Series and Clinical Considerations

**DOI:** 10.3390/jcm11040981

**Published:** 2022-02-13

**Authors:** Jakub Udzik, Paweł Jakubowski, Małgorzata Niekrasz, Adrian Barczyszyn, Miłosz Parczewski

**Affiliations:** 1 Department of Physiology, Pomeranian Medical University in Szczecin, Powstańców Wielkopolskich 72, 70-111 Szczecin, Poland; 2 Department of Neurosurgery and Neurotraumatology, Samodzielny Publiczny Wojewódzki Szpital Zespolony, Arkońska 4, 71-455 Szczecin, Poland; pjakubowski.med@gmail.com; 3 Department of Neurology and Cerebral Stroke, Samodzielny Publiczny Wojewódzki Szpital Zespolony, Akrońska 4, 71-455 Szczecin, Poland; mal.niekrasz@gmail.com; 4 Department of Radiology and Diagnostic Imaging, Samodzielny Publiczny Wojewódzki Szpital Zespolony, Arkońska 4, 71-455 Szczecin, Poland; barczyszyn@spwsz.szczecin.pl; 5 Department of Infectious Diseases, Tropical Diseases and Immune Deficiency, Pomeranian Medical University in Szczecin, 71-455 Szczecin, Poland; mparczewski@yahoo.co.uk

**Keywords:** COVID-19, covid-associated encephalopathy, COVID-19 neurological complications, neuroinfection

## Abstract

Neurological manifestations of the SARS-CoV-2 infection are present in up to 80% of the affected patients. While the majority of them is benign, in certain patients, viral replication in the central nervous system results in a severe disruption in cognitive function as well as basic life functions. In this case series, the authors present a detailed description of the three SARS-CoV-2 infection cases, which were all complicated by severe encephalopathy. Consecutive neurological status changes were described for each patient with detailed imaging and clinical sequelae. In the discussion, the authors highlight similarities in the course of the disease in presented patients, as well as common features in test results. An effective causal treatment could not be introduced in any of the patients, nor could the progression of the central nervous system (CNS) damage be stopped. The authors hope that the experiences they gathered will help to accelerate the diagnostic and therapeutic process in other patients with COVID-19-associated encephalopathy and result in introducing an effective treatment.

## 1. Introduction

Despite a recent discovery of the coronavirus disease (COVID-19), a wealth of data on the clinical manifestations related both to viral replication, clade variability, disease stage and patient comorbidities has been gathered thus far. Neurological symptoms, however less frequent, have been described as one of the key clinical features impacting this disease [1,2,3]. Involvement of the central nervous system related to the possible expression of viral proteins and its inflammatory and proapoptotic properties resulting in local inflammation and delayed synaptic signaling [4,5] has been well characterized. It should be stated that reports emphasize heterogeneity in COVID-19 neurological manifestations [6], which may range from mild symptoms such as headache and dizziness, psychomotor deceleration, memory impairment (including “brain fog”), anosmia, ataxia, speech disorders, neuralgia and to medium and severe complications such as neuropathic pain, muscular paresis and paralysis, epileptic seizures and coma—Table 1 [7,8,9].

Moreover, from a clinical perspective, vascular disorders (cerebral ischemia, thromboembolic events of the cerebral vasculature and cerebral bleeding), inflammatory disorders (mainly encephalitis and encephalopathy) and peripheral nerves disorders (Guillain–Barré syndrome, Miller–Fisher syndrome and neuralgia) [10,11,12,13,14] were previously observed. Thus far, severe encephalopathy associated with SARS-CoV-2 have been described only infrequently [15,16,17], with no clearly defined pathogenesis.

In this case series, the authors would like to present the cases of COVID-19 associated encephalitis and encephalopathy with unfavorable outcomes despite advanced differential diagnostics and therapeutic efforts. The authors would like to lay the ground for further studies on the brain-related CNS pathology associated with SARS-CoV-2 infection.

## 2. Clinical Cases

### 2.1. Patient 1

A 71-year-old female of Caucasian ethnicity was admitted with speech disorders and allopsychic orientation disturbances. Approximately 2–3 days prior to admission, gastrointestinal symptoms (vomiting, nausea and diarrhea) were observed. Medical history included arterial hypertension, type 2 diabetes and hyperthyroidism. Upon examination, only psychomotor decelerations with mild confusion were observed. SARS-CoV-2 infection was confirmed with molecular testing; in laboratory analyses, moderate increases of C-reactive protein (52.61 mg/L with the upper limit value of 5 mg/L) with no other significant laboratory abnormalities were noted. Chest computed tomography (CT) revealed mild, diffuse lesions in both lungs, consistent with interstitial pneumonia, while in the CT scan of the head, minor hemorrhagic foci of the supratentorial white matter, consistent with chronic ischemic lesions in small vessel disease, were present. Cortical and subcortical atrophy was also noted, with adequate dilatation of the cerebrospinal fluid (CSF) spaces. Based on the clinical and laboratory data above, a presumptive diagnosis of encephalitis in patient with COVID-19 was conducted. Treatment with dexamethasone (4 mg/day for 3 days with subsequent dosage increase to 16 mg/day for another 7 days), azithromycin (500 mg/day for 7 days) and enoxaparin (60 mg/day for 12 days) was initiated. 

During hospitalization, non-convulsive epileptic seizures were diagnosed based on paroxysmal activity in EEG examination, with the tendency to generalize in the left frontotemporal area. Levetiracetam (500 mg/day) was introduced into the treatment. As in the subsequent days, a transient decrease in right upper limb muscular strength (4/5 on the Lovett scale [18]) was observed, ceftriaxone (4 g/day for 7 days) and acyclovir (2 g/day for 9 days) were introduced into the treatment. Fluctuations in the qualitative and quantitive consciousness were observed, with transient time and location disorientation and variable verbal contact. Due to clinical deterioration in pulmonary parameters, progression of inflammatory lesions in the lungs and laboratory features of cytokine storm, an anti-interleukin receptor 6 therapy with tocilizumab was administered, supplemented with meropenem (6 g/day for 10 days) and methylprednisolone (1 g/day for 10 days).

**Figure 1:**
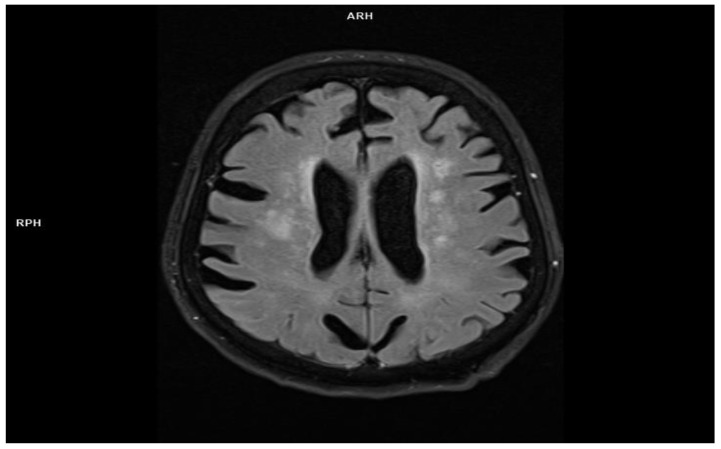
Cortical-subcortical atrophy with the diffuse hyperintense areas of the white matter (small vessel disease)—Flair sequence (MRI imaging).

MRI examination of the brain (Flair and T2 sequences) revealed multiple, speckled, hyperintense foci in the supratentorial white matter, located deep in the brain and around the ventricles (Figure 1).

**Figure 2:**
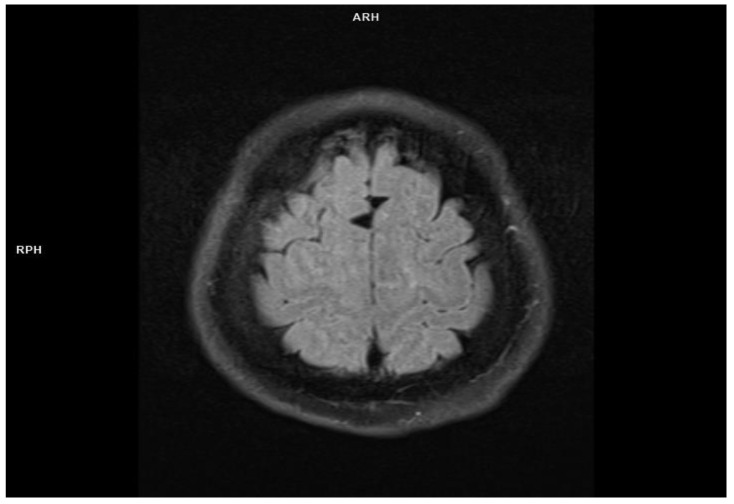
Bilateral shallowed sulci on the convexities of the brain—Flair sequence (MRI imaging).

The foci had a tendency to confluence, showed no contrast enhancement and no diffusion restriction and were consistent with small vessel disease. Stage 2 leucoaraiosis on the Fazekas scale [19] was also present. Shallow sulci with blurred edges were observed on the convexities of the brain (Figure 2)—inflammatory etiology was suspected.

In the other regions of the brain, sulci were significantly dilated mostly within the temporal and frontal lobes but also within the occipital lobes. The image was consistent with advanced cortical-subcortical atrophy. Ventricles were symmetrical and moderately dilated, while perivascular spaces were centrally enlarged. 

After 14 days, the patient’s respiratory efficiency improved (capillary blood saturation was 93% without the oxygen delivery). Despite the normalization of inflammatory parameters in the following days and introduction of therapy with cerebrolysin (8.61 g/day for 10 days) and amantadine (200 mg/day for 6 days), the patient’s mental state and quantitative consciousness were systematically deteriorating, with horizontal multidirectional nystagmus, bilateral abducens nerve (VI) palsy and roving eye movements, with a fatal outcome on the 41th day from admittance.

During in-hospital stay, three lumbar punctures were performed with CSF sampling (on the 3rd, 16th and 34th day)—Table 2. Slightly increased protein and glucose concentrations (maximal protein concentration was 75.1 mg/dL and 79 mg/dL for glucose) and no cellular abnormalities were noted, with moderate blood–brain barrier damage and no anti-NMDAR, AMPAR1, AMPAR2, CASPR2, LGI1 or DPPX antibodies. The increased CSF protein level was due to an intrathecal synthesis of albumins and class G immunoglobulins. Furthermore, molecular infectious disease CSF testing for adenoviruses; enteroviruses; Varicella Zoster Virus (VZV); Human Herpesvirus 6 (HHV-6); Human Herpesvirus 7 (HHV-7); Epstein–Barr Virus (EBV); Herpes Simplex Virus 1 (HSV-1); Herpes Simplex Virus 2 (HSV-2); human Cytomegalovirus (hCMV); Human Parechovirus; Parvovirus B19; and Human polyomavirus 2 (JCV) as well as borrelia antibody proved negative. Finally, negative autoantibody results relative to anti-DFS70, c-ANCA, p/ANCA, p/MPO, ANA, anti-NMDA and anti-neuronal antibodies (Amphiphysin, CV2.1, Ma2/Ta, Ri, Yo, Hu) were also obtained. 

### 2.2. Patient 2

A 71-year-old Caucasian male was admitted with impaired verbal contact, dyspnoea and fever with SARS-CoV-2 infection confirmed two days previously. In his medical history, the patient had cerebral stroke (3 years ago) with left hemiparesis, type 2 diabetes, dyslipidemia, obesity and smoking; however, the patient used to be self-sufficient, with full logic and verbal contact. The patient’s general condition on admittance was serious; he exhibited sensory-motor aphasia and required oxygen delivery. The ground-glass opacity typical for COVID-19 pneumonia was present in the chest CT scan. The presence of an 8 mm intracerebral hematoma in the left thalamus was noted on head-computed tomography. Inflammatory parameters were moderately elevated (CRP—53.79 mg/L, IL-6—62.1 pg/mL with the upper value limit of 7 pg/mL) with high D-dimer concentrations (20,777 μg/L with the upper value limit of 500 μg/L). 

Intracerebral hemorrhages in a patient with SARS-CoV-2 pneumonia, concomitant aphasia and suspected pulmonary artery thrombosis were diagnosed and treated with azithromycin (500 mg/day for 4 days) and ceftriaxone (2 g/day, after 24 h the dosage was increased to 4 g/day and continued up to the 5th day of hospitalization) and dexamethasone (8 mg/day for 8 days). After confirming the pulmonary artery, thrombosis dalteparin was introduced into the treatment (15,000 units/day for 7 days). Unfortunately, aphasia persisted and it was only temporarily possible to establish a simple logical contact with the patient, despite the addition of acyclovir (2 g/day for 5 days) and cerebrolysin (6.46 g/day for 6 days) into therapy. 

Within the first week of treatment, the patient’s condition deteriorated, with tetraparesis and meningeal signs. Respiratory efficiency, however, improved after anticoagulation treatment and was stable until the seventh day of hospitalization. In the EEG, basic electrical activity of the brain was present in the form of theta waves with a frequency of 5–7 Hz, with concomitant beta activity in frontal leads. During the examination, multiple, generalized sharp wave discharges were registered. The examination report suggested encephalitis while, in the follow-up head CT, a lack of hematoma in the left thalamus that was discovered in the first examination was noted. An MRI was not performed due to clinical instability. The patient continued to deteriorate with a decrease in CRP (23.01 mg/L) and IL-6 (43.1 pg/mL), hyperkalemia (6.97 mmol/L) and hypernatremia (152.5 mmol/L), clinical tetraplegia and an impaired pupillary reflex followed by a respiratory failure and death on the eighth day during in-hospital treatment. 

Here, CSF was examined on the second day from admittance with increased glucose (123 mg/dL), chlorine ions (135.8 mmol/L) and protein (72.1 mg/dL) levels—Table 2. Number of leukocytes was slightly elevated (7 cells/μL). The serological and molecular tests were negative for HIV, HBV, HCV, CMV and toxoplasmosis. 

### 2.3. Patient 3

A 64-year-old Caucasian male with a history of peripheral neuropathy with concomitant abnormal gait and neuropathic pain (treated with 900 mg of gabapentin a day) was admitted with agitation, inexpedient motoric behaviors and bradyphrenia accompanied by cough and fever, which started seven days previously. 

On examination temporary disorientation, confusion and inexpedient limb movements were observed. Similarly to the previous cases, diffuse lesions in both lungs (“the ground-glass opacity”), consistent with an interstitial pneumonia, were observed, with moderate elevations of inflammatory parameters (CRP—59.35 mg/L, IL-6—26.3 pg/mL). In the head CT scan, only areas of periventricular leukoaraiosis were present. Initially, the patient was treated with a standard dose of remdesivir, dexamethasone (24 mg/day for 7 days), ceftriaxone (4 g/day for 12 days), levofloxacin (1 g/day for 12 days), acyclovir (1.5 g/day for 10 days) and enoxaparin (120 mg/day for 12 days). Due to clinical respiratory deterioration with psychomotor agitation, convalescent plasma with an interval of 24 h was administered, but no clinical improvement related to the psychomotor function was noted despite the decrease in inflammatory parameters. In the following days, opisthotonus developed with loss of the verbal contact, tetraparesis with the right-side predominance, upper extremities rigidity and no tendon reflexes in the lower limbs. The pyramidal signs were absent. Acyclovir (250 mg/day for 16 days), cerebrolysin (8.61 g/day for 7 days), amantadine (200 mg/day for 6 days) and methylprednisolone (1 g/day for 5 days) were introduced into the therapy and supplemented with analgosedation with morphine (40 mg/day) and diazepam (30 mg/day). The patient required a mild oxygen support (capillary blood saturation was 89% with no oxygen delivery). In the EEG examination, a dominating low-voltage fast activity was present, with transitory theta waves discharges (5.5–6 Hz, up to 30 μV) in all leads. 

**Figure 3:**
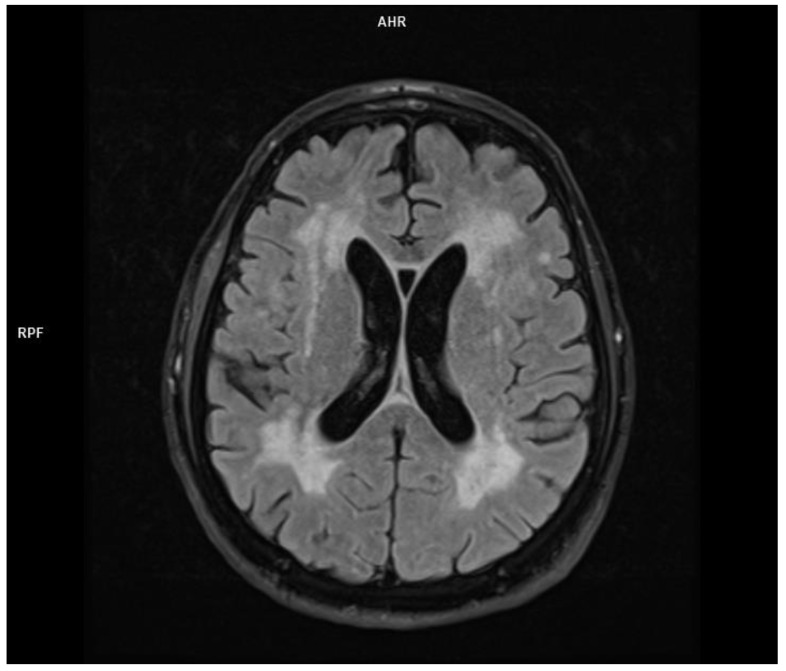
Moderate cortical atrophy and supratentorial ventricular system dilatation—Flair sequence (MRI imaging).

**Figure 4:**
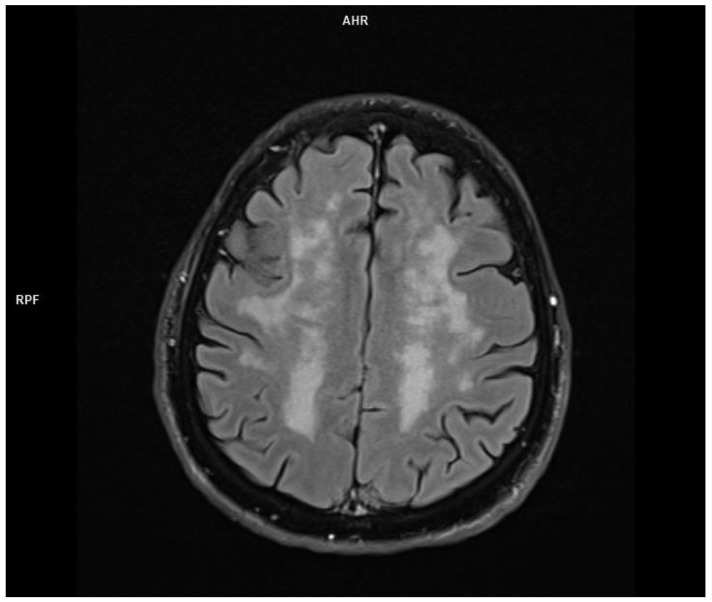
Diffuse hyperintense areas of the subcortical white matter—Flair sequence (MRI imaging).

In the brain MRI, multiple foci and diffused areas of the hyperintense lesions in the supratentorial white matter in the Flair and T2 sequences (Figure 3 and Figure 4) were noted. 

**Figure 5:**
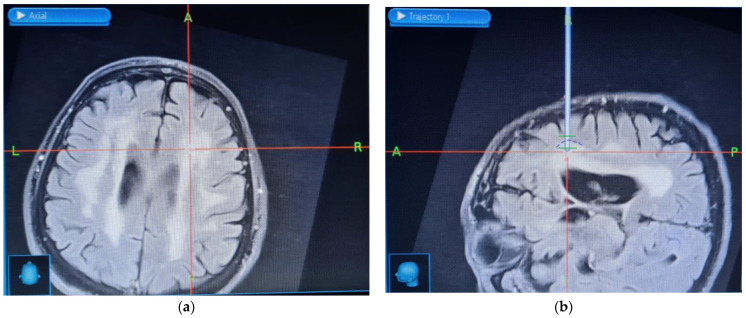
Stereotactic neuronavigation images: (**a**) the suspected area of brain tissue inflammation. The axes cross in the spot from which the sample was taken within the right frontal lobe; (**b**) the image of the border area between a healthy tissue and the lesions (the border is marked with a dashed blue line). The borders of the biopsy frame are marked in green.

The lesions were consistent with small vessel disease, with no contrast enhancement or diffusion restriction. There was a 4 mm cavity present in the deep brain structures of the left side, consistent with focal ischemia. A few similar cavities were present within the pons. The DWI sequence showed no acute post-infarction foci, and there were no signs of the mass effect. A moderate cortical atrophy was noted within brain convexities. In light of the patient’s severe condition and a lack of further diagnostic alternatives, a decision was made to perform a brain biopsy. Using stereotactic neuronavigation, four brain tissue samples were taken from the right frontal area, using variable sampling depths to include the borderline area between a healthy tissue and the lesions described in the MRI examination report (Figure 5). 

However, in the histopathological examination of the brain tissue samples, there was no inflammatory infiltration, only a focal hemorrhaging was present. Following the procedure, further neurological progression was noted with a loss of pharyngeal reflexes, and for this reason, human immunoglobulins were started (4 g/day). The treatment was discontinued within a few hours as the patient’s temperature rose, suggestive of an adverse reaction to immunoglobulins. The patient’s respiratory efficiency deteriorated on the 40th day of hospitalization (he required a high flow oxygen supplementation), with a considerable leukocyte count increase (26.42 K/μL). Despite further treatment with extended spectrum antibiotics (meropenem (6 g/day) and vancomycin (3 g/day), the patient died on the 44th day of hospitalization.

Here, four lumbar punctures were performed in total (on the 2nd, 7th, 13th and 28th day)—Table 2—with the predominant abnormality on all being increased CSF glucose levels with no cytological abnormalities. CSF was also examined for the indicators of the Creutzfeldt–Jakob disease and genetic material of the adenoviruses, enteroviruses, Varicella Zoster Virus (VZV), Human Herpesvirus 6 (HHV-6), Human Herpesvirus 7 (HHV-7), Epstein-Barr Virus (EBV), Herpes Simplex Virus 1 (HSV-1), Herpes Simplex Virus 2 (HSV-2), human Cytomegalovirus (hCMV), Human Parechovirus, Parvovirus B19, Human polyomavirus 2 (JCV) and the SARS-CoV-2 virus—all results proved negative. CSF and serum were both negative for IgG and IgM anti-borrelia antibodies (in Western-blot test). Finally, the presence of the following autoantibodies was excluded in CSF: NMDAR, AMPAR1, AMPAR2, CASPR2, LGI1 and DPPX. Similarly to the two cases presented above, all other virological serological testing and autoantibody assays proved negative. The concentration of the immunoglobulin classes A, G and M in the serum was normal. Moreover, the IgG subclasses’ (IgG1—IgG4) concentration was normal. No oligoclonal bands were detected in the blood or CSF.

## 3. Discussion

Severe COVID-19 associated encephalopathy is usually described in patients in whom the SARS-CoV-2 infection resulted in a critical organ failure (in most cases it is a respiratory failure) [20,21]. In the presented case series, encephalopathy was one of the earliest symptoms of the COVID-19 infection (it developed prior to a respiratory instability) and it remained a leading clinical problem for those patients during the entire treatment process. Unlike in the other scientific investigations, in this instant, the timeline of events demonstrates that encephalopathy was the primary cause of the subsequent respiratory failure and clinical state deterioration, and that it was an initial cause of death in those patients. Patient 1 did not require oxygen delivery after the 14th day of the in-hospital stay, whereas her neurological condition was systematically deteriorating. In patient 2, respiratory efficiency improved after the anticoagulation treatment and remained stable until the day prior to his death, whereas his neurological condition deteriorated rapidly from the beginning of hospitalization. On the day of his death, in addition to the respiratory failure, patient 2 exhibited not only severe neurological deficiencies but also dyselectrolytemia and severe hypotension, which did not respond to any treatment and is suggestive of damage to the deep brain structures such the hypothalamus and brain stem. In patient 3, systematic neurological deterioration persisted in spite of the fact that he required mild to moderate oxygen support throughout most of his in-hospital stay.

The common denominator of those three cases is that the neurological deterioration was continuous and progressive, and it did not correlate with the oxygen demand. None of these patients developed respiratory failure before developing serious encephalopathy.

The common features of the presented three cases of the COVID-19 associated encephalopathy were normal CSF cell count, lack or mild elevation of protein in the CSF with slightly elevated glucose levels, diffuse inflammatory lesions in the white matter of the brain and low to mild increases in the CRP activity. EEG examination revealed a paroxysmal activity in patient 1, whereas patients 2 and 3 presented slow wave brain activity consistent with the picture of encephalitis. In all cases, no significant central nervous disease prior to COVID-19 was observed. In all cases, neurological status alteration was associated with the SARS-CoV-2 infection and lack of inflammation in the CSF. 

The mechanism of the nervous system damage in COVID-19 has not been fully explained yet, although many attempts are made. Certainly, pathology is multifactorial, complex and involves neuronal damage by the expressed viral proteins and use of the angiotensin converting enzyme-2 receptor (ACE-2 receptor) [22], which is also present within the nervous system, namely in the neurons and glial cells [23]. The animal studies showed that the ACE-2 receptor is present within the entire brain and not only in perivascular spaces such as the olfactory bulb and cortex as well as deep brain structures [24,25]. To infiltrate central nervous system, the virus crosses the blood–brain barrier using ACE-2 receptor in the endothelial cells of the brain vessels and pericytes [26,27]. Within the neurons, the virus alters metabolic pathways and deregulates cellular activity by impairing the cell’s antioxidant defense mechanisms and compromising DNA repair [28]. This results in an increased presence of the reactive oxygen species (ROS) within the neurons and eventually results in apoptosis [29,30,31].

Another possible cause that might explain nervous system damage in COVID-19 is the presence of anti-neuronal antibodies and autoantibodies, which attack the host’s cells, resulting in permanent changes in the central nervous system [32]. Reports on the association between SARS-CoV-2 infection and neurodegenerative processes were published with the suggested underlying autoimmune mechanism [33]. Within these degenerative diseases, the Guillain–Barré syndrome (GBS), the Miller–Fisher syndrome (MFS) and the Acute Disseminated Encephalomyelitis (ADEM) are described [34,35,36]; however, in the presented cases, no autoantibodies suggesting the above diseases were present neither in blood nor in CSF. 

CSF examination is one of the basic tools in the diagnostics of the neurological manifestations of COVID-19 [37,38]. Apart from the above-mentioned anti-neuronal antibodies and autoantibodies, the main markers of SARS-CoV-2 infection in the CSF are protein concentration, leukocyte count and the presence of the viral genetic material. In the majority of COVID-19 cases described so far, the RT-PCR test of the CSF was negative for the SARS-CoV-2 genetic material [39]. Some of these patients had elevated protein concentration and leukocyte count in the CSF. It is consistent with observations made in this study. 

Two patients in this study had elevated protein concentration in the CSF. In none of these patients the SARS-CoV-2 genetic material or a significant cell count were found in the CSF. This supports the thesis that neurological manifestations and neurodegenerative processes in COVID-19 have a complex pathological background and are not directly related to SARS-CoV-2 presence in CSF. Discovering the exact pathomechanism of neuroinvasion or neuroinflammation in this disease could aid the understanding of the pathophysiology of neurological manifestations of COVID-19 [40,41,42,43]. Imaging examinations are a key diagnostic tool, with MRI imaging being the preferred method. There are multiple reports and study cases describing pathological changes present in MRI examination in patients with COVID-19, including ischemic changes (TIA, ischemic stroke and small vessel disease), hemorrhagic changes (hemorrhagic stroke and subarachnoid hemorrhage) and inflammatory changes (meningitis, encephalitis and acute disseminated encephalomyelitis) [44,45,46,47,48,49].

Two of the three patients described in this study had the brain MRI examination result. In patient 1, the MRI picture indicated inflammatory etiology, whereas in patient 3 the described lesions were consistent with small vessel disease. All patients clinically manifested the symptoms of progressing encephalopathy, which did not respond to any kind of treatment and eventually resulted in the death of the patients. The clinical picture of COVID-19-associated encephalopathy in these patients is consistent with the scientific reports of other researchers and it embodies the need to develop new diagnostic methods for neuro-COVID-19 [50,51,52,53,54,55,56,57,58,59,60,61,62,63,64].

The above description of the medical cases combines clinical data with additional tests results (imaging, laboratory and functional examinations). It shows the range of the applied diagnostics and the effects of the applied treatment. The authors hope that the experiences they gathered will help to accelerate the diagnostic and therapeutic process in other patients, potentially resulting in earlier diagnosis and introducing a more effective treatment.

## 4. Conclusions

SARS-CoV-2 infection can lead to the development of a severe encephalopathy, with a clinical picture of encephalitis and poor treatment responses. Discovering and explaining the pathomechanism that results in central nervous system damage in COVID-19 and introducing new diagnostics methods are crucial for developing a fully effective treatment strategy for the patients infected with the novel coronavirus.

## Figures and Tables

**Table 1:** Neurological symptoms in patients with COVID-19.

Mild	headache
	dizziness
	anosmia
Moderate	psychomotor deceleration and memory impairment (including “brain fog”)
	ataxia
	speech disorders
	neuralgia and neuropathic pain
Severe	muscular paresis and paralysis
	epileptic seizures
	coma

**Table 2:** Results of the biochemical and cytological analysis of the consecutive CSF samples in all patients.

	Parameters	LP 1	LP 2	LP 3	LP 4
Patient 1	Glucose:	79	77	47	X
	Cl^−^:	127.9	121.7	119.6	X
	Protein:	66.5	75.1	72.3	X
	WBC:	2	1	1	X
	Macrophages:	0	0	1	X
Patient 2	Glucose:	123	X	X	X
	Cl^−^:	135.8	X	X	X
	Protein:	72.1	X	X	X
	WBC:	7	X	X	X
	Macrophages:	0	X	X	X
Patient 3	Glucose:	76	93	85	78
	Cl^−^:	127.9	137.1	127.1	121.8
	Protein:	24.7	39.9	38.2	22.7
	WBC:	2	4	5	2
	Macrophages:	0	0	0	0

Legend: Cl^−^—chlorine ions concentration in the CSF (mmol/L); Glucose—glucose concentration in the CSF (mg/dL); LP (1–4)—consecutive lumbar punctures performed in each patient; Macrophages—number of macrophages identified in the CSF (cells); Protein—protein concentration in the CSF (mg/dL); WBC—white blood cells count in the CSF (cells/uL); X—further lumbar punctures were not performed in this patient. Red color signifies that the result was not within the referential range. Referential values: glucose: 40–70 mg/dL; Cl^−^: 115–130 mmol/L; protein: 15–65 mg/dL; WBC: 0–5 cells/uL; macrophages: 0 cells.

## Data Availability

Not applicable.

## Abbreviations

NMDAR—N-methyl-D-aspartate receptors; AMPAR1—α-amino-3-hydroxy-5-methyl-4-isoxazolepropionic acid receptor 1; AMPAR2—α-amino-3-hydroxy-5-methyl-4-isoxazolepropionic acid receptor 2; CASPR2—Contactin-associated protein-like 2; LGI1—Leucine-rich glioma-inactivated 1; DPPX—Dipeptidyl-peptidase–like protein-6.
